# The effect of Perilla (*Perilla frutescens)* leaf extracts on the quality of surimi fish balls

**DOI:** 10.1002/fsn3.1049

**Published:** 2019-05-03

**Authors:** Yueliang Zhao, Hongyun Kong, Xu Zhang, Xiaoqian Hu, Mingfu Wang

**Affiliations:** ^1^ College of Food Science and Technology Shanghai Ocean University Shanghai China; ^2^ Laboratory of Quality and Safety Risk Assessment for Aquatic Products on Storage and Preservation (Shanghai) Ministry of Agriculture Shanghai China; ^3^ School of Biological Sciences The University of Hong Kong Hong Kong China

**Keywords:** antioxidant, perilla leaf extracts, quality evaluation, surimi fish ball

## Abstract

The effects of *Perilla frutescens* leaf extract (PLE) on the quality of surimi fish balls were investigated in the present study. Firstly, the extract was prepared by solvent extraction using 95% ethanol. Then, the phenolics in the extract were analyzed by instrumental analysis. The total phenolic content in the PLE was found to be 14.51 mg gallic acid equivalent (GAE)/g dry weight (DW). The amount of caffeic acid, ferulic acid, rosmarinic acid, quercetin, and apigenin, determined by high‐performance liquid chromatography (HPLC), was 4.80, 5.10, 2.95, 6.46, and 3.93 mg/g DW, respectively. Furthermore, the PLE was found to show high free radical scavenging activity toward DPPH and ABTS radicals with IC_50_ values of 12.15 and 7.26 μg/ml, respectively. When PLE was fortified into surimi fish balls at 0.03% and stored at 4°C, it was found to slow down lipid and protein oxidation during storage of surimi fish balls as evidenced by the significant reduction in TBARS values and protein carbonyl contents (*p* < 0.05). PLE (0.03%) also decreased the formation of total volatile basic nitrogen (TVB‐N) and inhibited the growth of *E*. *coli* compared with the control group (*p* < 0.05). In addition, the overall acceptability of PLE‐added (0.03%) samples was higher than control samples during the storage process (*p* < 0.05) by sensory analysis. Overall, PLE have the potential to be used as a natural food additive to improve the shelf life and sensorial qualities of surimi fish ball.

## 1 INTRODUCTION

Surimi is an inexpensive source of protein and useful ingredient for manufacturing a wide array of fish‐based food products such as fish ball, imitation crab meat, fish sausage, paupiette, and breaded fish stick. Surimi‐based products have become increasingly popular in recent years due to their unique textural properties, low fat, low cholesterol, and high nutritional value (Yousefi & Moosavi‐Nasab, 2014). In 2015, the production of surimi‐based products has reached 1.5 million tons in China (An, You, Xiong, & Yin, 2018). Due to the special method of handling and nutritional characteristics, lipid oxidation and microbial growth have been considered two important factors leading to quality deterioration and shelf life reduction in surimi products during refrigerated storage.

Lipid oxidation in meat products is the major cause of their quality deterioration. Polyunsaturated fatty acids are prone to oxidation which produces secondary oxidative compounds, such as hexanal, heptanal, pentanal, and octenal. These volatile aldehydes mostly contribute to the undesirable flavors described as warmed‐over flavor (WOF) develops in refrigerated cooked meat products (Kim, Li, Lim, Kang, & Park, 2016). Lipid oxidation can also negatively affect other quality parameters such as flavor, color, texture, and even the nutritional value of meat products (Devatkal, Narsaiah, & Borah, 2010). Total lipids of surimi‐based products contained relatively high proportion of polyunsaturated fatty acids susceptible to oxidation (Hosseini Shekarabi, Hosseini, Soltani, Kamali, & Valinassab, 2014). Thus, it is necessary to develop strategies to inhibit lipid oxidation of surimi‐based products during storage. In addition, ready‐to‐eat meats can be contaminated with various foodborne pathogens under refrigerated storage conditions, which may produce undesirable changes in food quality and cause foodborne illnesses (Rouger, Tresse, & Zagorec, 2017). For example, *E. coli* O157:H7 is a food‐related pathogen that can cause hemorrhagic uremic syndrome and hemorrhagic uremic colitis. It commonly grows on meat products in low temperature and acidic conditions and produces undesirable quality changes in meats (Ahn, Grün, & Mustapha, 2007). Similar to meat products, surimi‐based products can also be contaminated with microorganisms during storage. Thus, enhanced oxidative stability and reduced microorganisms are required for maintaining the quality and safety of surimi‐based products.

Synthetic antioxidants such as butylated hydroxyanisole (BHA) and butylated hydroxytoluene (BHT) have been used extensively to preserve meat products because of their low cost and high efficiency (Kumar, Yadav, Ahmad, & Narsaiah, 2015). However, recently, it has been realized that these synthetic additives may have toxic effects. There is an increasing demand for natural antimicrobials and antioxidants. *Perilla frutescens*, belonging to the family Lamiaceae, is a dual‐purpose plant widely cultivated in China, Thailand, and Southeast Asia which can be used for food and medicine (Zhou et al., 2014). It is deemed as a cate in the United States, Japan, Canada, and Russia, mainly used in sushi, garnish, and soup. *Perilla frutescens* organs have been found to contain abundant natural molecules, including flavonoids, phenolic acids, triterpenes, carotenoids, and essential oils. *Perilla frutescens* leaf extracts have shown antioxidant, antibacterial, anti‐inflammatory, and antitumor effect (Skowyra, Falguera, Azman, Segovia, & Almajano, 2014). Recently, our group found that essential oil extracted from *Perilla frutescens* leaf can improve the quality of surimi‐based food (Kong, Zhou, Hu, Wang, & Wang, 2018). However, whether the Perilla (*Perilla frutescens*) leaf extracts, being a rich source of healthy natural antioxidants with unique flavor, could replace synthetic antioxidants to extend the shelf life and enhance the quality of surimi‐based food during storage remains unknown.

The purpose of this project was to study the effect of *Perilla frutescens* leaf extracts (PLE) on physicochemical properties (lipid and protein oxidation and total volatile basic nitrogen), microbial quality, and sensory characteristics of surimi fish ball stored at 4°C for 12 days. This study provided evidence for the use of *Perilla frutescens* leaves as food additives to improve the quality of surimi‐based foods.

## 2 MATERIALS AND METHODS

### 2.1 Preparation of *Perilla frutescens* leaf extract (PLE)

*Perilla frutescens* leaves were purchased from local markets in Shanghai. After washing with distilled water, the leaves were dried at room temperature. The leaves were then weighted and grounded, and the powder was passed through a 70‐mesh sieve and stored in darkness, at room temperature. 10 g of the filtered powder was immersed with 95% ethanol (60 ml) and extracted with reflux condensation for 1 hr. The homogenate was filtered through filter paper (Whatman No. 4), and the residue was re‐extracted twice with 30 ml of 95% ethanol as described above. The combined filtrate was evaporated to a minimum volume on a rotary evaporator at 40°C under vacuum. After that, AB‐8 macroporous resin was applied to remove sugars in the extract. The extract obtained then was concentrated by rotary vacuum evaporation and freeze‐dried. The dried extracts were stored at 4°C for future use.

### 2.2 Total phenolics of PLE

The total phenolic content of *Perilla frutescens* leaf extracts was determined by Folin–Ciocalteu reagent assay (Ozsoy, Can, Yanardag, & Akev, 2008). Briefly, 1 ml of the extract (1 mg/ml) was mixed with 1 ml of Folin–Ciocalteu reagent and 3 ml of 2% sodium carbonate solution. The mixture was vortexed and kept in the dark for 1 hr. The absorbance of the mixture was then measured at 760 nm on a UV‐2300 ultraviolet spectrophotometer (Shanghai Techcomp Ltd.). Gallic acid was used to establish standard curve. The total phenolic content was expressed as mg gallic acid equivalent (GAE)/g dry weight (DW).

### 2.3 The polyphenolic profile of PLE

The polyphenolic profile of *Perilla frutescens* leaf extracts was determined using HPLC (Waters Corp.) with a diode array detector (DAD). *Perilla frutescens* leaf extracts were isolated on a C18 column (5 μm, 4.6 × 250 mm, Waters). The mobile phase for HPLC analysis was composed of acetonitrile (solvent A) and 0.1% phosphoric acid (solvent B) of the following gradients: 0 min, 5% A: 95% B; 3 min, 5% A: 95% B; 6 min, 10% A: 90% B; 8 min, 30% A: 70% B; 9 min, 35% A: 65% B; 11 min, 40% A: 60% B; 12 min, 50% A: 50% B; 15 min, 65% A: 35% B; 20 min, 75% A: 25% B; 25 min: 90% A: 10% B; and 28 min, 5% A: 95% B. DAD was set at 280 nm. The flow rate was 1 ml/min. 10 µl of *Perilla frutescens* leaf extracts was used for HPLC analysis after filtration through a 0.45‐μm filter. Peak identification was performed by comparing the retention time and ultraviolet absorption spectrum of the eluting peaks with those of the polyphenol standards.

### 2.4 Antioxidant activities of PLE

The DPPH radical scavenging activity of *Perilla frutescens* leaf extracts was determined according to previous studies with slight modifications (Zhang, Wu, & Guo, 2016). Briefly, 0.5 ml of *Perilla frutescens* leaf extracts was added to 1 ml of DPPH (50 mg/L) in methanol. The mixture was shaken vigorously and left to stand for 20 min at room temperature in the dark. The absorbance was measured at 519 nm by using an UV‐2300 ultraviolet spectrophotometer (Shanghai Techcomp Ltd.). The DPPH radical inhibition rate of the *Perilla frutescens* leaf extracts was calculated as follows:

DPPH radical scavenging activity (%) = $\frac{A_{\text{blank}}-A_{\text{sample}}}{A_{\text{blank}}}\times 100\%$

ABTS radical scavenging activity of *Perilla frutescens* leaf extracts was measured by the ABTS cation decolorization assay as described by Skowyra et al. (2014). Briefly, a stock solution of ABTS•+ was prepared by mixing 7.4 mmol/L ABTS solution with 2.45mmol/L potassium persulfate and allowing the mixture to stand in the dark at for 12 hr at room temperature before use. The ABTS•+ working solution was prepared by dilution of the stock solution of ABTS•+ with ethanol to achieve an absorbance of 0.70 ± 0.02 at 734 nm. 0.2 ml of *Perilla frutescens* leaf extracts and 0.8 ml of ABTS•+ working solution were mixed for 6 min, and the absorbance was measured at 734 nm using an UV‐2300 ultraviolet spectrophotometer (Shanghai Techcomp Ltd.). For the blank, 0.2 ml of PBS was used. The ABTS radical inhibition rate of the *Perilla frutescens* leaf extracts was calculated as follows:

ABTS radical scavenging activity (%) = $\frac{A_{\text{blank}}-A_{\text{sample}}}{A_{\text{blank}}}\times 100\%$

### 2.5 Preparation of surimi fish ball

The surimi fish ball was prepared according to our previous study with slight modifications (Kong et al., 2018). Briefly, *Argyrosomus argentatus* surimi was purchased from Shishi Zhengyuan Aquatic Co., Ltd. The frozen *Argyrosomus argentatus* surimi stored at −20°C was thawed using floating water for about 30 min and then mixed with *Perilla frutescens* leaf extracts (0, 0.1, 0.3 g/kg). The mixture was chopped using a silent cutter at low speed for 6 min. The surimi was then added with salt (2.5 g/kg) and chopped for another 8 min. The surimi paste obtained was placed into molds to give the fish balls. After that, the surimi was subjected to gelatinization for 2 hr at 37°C and then boiled at 95°C for 15 min, followed by cooling in ice‐cold tap water for 10 min. Finally, the surimi fish balls were air‐packed and stored at 4°C for 12 days. The food quality parameters were measured every three days.

### 2.6 Thiobarbituric acid reactive substance (TBARS) analysis

Lipid oxidization was monitored by measuring TBARS value according to previous study with slight modifications (Erkan & Özden, 2008). Briefly, TBARS in sample (5.0 g) was extracted with chilled 20% (w/v) trichloroacetic acid (TCA) (45 ml) for 5 min. The mixture was kept at room temperature for about 1h and then centrifuged at 4,000 *g* for 15 min. 5 ml of supernatant was added to 5 ml of 0.02 M TBA, and it was heated at 95°C for 30 min. After cooling to room temperature using tap water, the absorbance was measured on a UV‐2300 ultraviolet spectrophotometer (Shanghai Techcomp Ltd.) at 532 nm. The TBARS value was calculated as mg of malondialdehyde (MDA) per kg of sample.

### 2.7 Protein carbonyl content analysis

The protein carbonyl content was determined according to Xiao and others with slight modifications (Xiao, Zhang, Lee, Ma, & Ahn, 2011). Briefly, 0.1 g of sample was homogenized with 0.9 ml pyrophosphate buffer (pH = 7.4) containing 2.0 mM Na_4_P_2_O_7_, 2.0 mM MgCl_2_, 100 mM KCl, 10 mM tris‐maleate, and 2.0 mM EGTA. Two equal volumes of homogenates (0.1 ml) were precipitated with 1 ml of 10% TCA. After centrifugation at 12,000 *g* for 5 min, one pellet was mixed with 0.4 ml 10 mM 2,4‐dinitrophenylhydrazine (DNPH) dissolved in 2 M HCl for carbonyl concentration measurement and the other was incubated with 0.4 ml 2 M HCl for protein concentration quantification. Both mixtures were kept in dark for 1 hr at room temperature. After that, the proteins were further precipitated with 1 ml of 10% TCA. The pellets were washed with 1 ml of the ethanol/ethyl acetate mixture (1:1 v/v) for three times and dissolved in 1.25 ml of 6 M guanidine hydrochloride in 20 mM potassium phosphate buffer and kept at 37°C for 15 min. Protein concentration was determined using a spectrophotometer at 280 nm with BSA as a standard. The carbonyl concentration was measured by reading the absorbance at 370 nm and calculated as nmol carbonyl/mg of protein using the adsorption coefficient of 22.0 mM^−1^ cm^−1^.

### 2.8 Total volatile basic nitrogen (TVB‐N) analysis

TVB‐N content was measured by distillation after addition of MgO (0.2 g) to the samples (2.0 g) in a distilling flask of Kjeldahl (Kjeltec 8400, FOSS, Hiller) (Yi et al., 2011). TVB‐N content was expressed as mg of nitrogen per kg of sample.

### 2.9 Microbial analysis

The total plate count and the populations of E. coli were determined according to previous study with slight modifications (Kong et al., 2018). Briefly, 3 g samples were homogenized for 3 min in 27 ml sterile saline (0.85%, w/v). The supernatant was serially diluted (1:10) in 0.85% saline solution. Sample dilutions (1 ml) were spread on the plates. The total plate count on nutrient agar was determined after incubation at 37°C for 48 hr. The populations of E. coli were determined on violet red bile agar after incubation at 37°C for 24 hr. Microbial colonies were expressed as CFU (colony‐forming units)/g sample.

### 2.10 Sensory evaluation

The effect of PLE on the sensory characteristics of the samples was evaluated by a trained panel of six members. The panelists were scored for various sensory attributes, including appearance, flavor, texture, taste, and overall acceptability on a 5‐point hedonic scale, where 1 means very poor, 2 means poor, 3 means fair, 4 means good, and 5 means excellent (Wei et al., 2014).

### 2.11 Statistical analysis

Experiments were performed at least three times, independently (*n* = 3). The data were presented as mean ± *SD*. Statistical analyses were conducted using SPSS for Windows version 17.0 (SPSS Inc.). The least significant difference (LSD) at the 5% confidence level was used for comparing treatments.

## 3 RESULTS AND DISCUSSION

### 3.1 The content of phenolic compounds in *Perilla frutescens* leaf extracts (PLE)

In the present study, the total phenolic content in the PLE was 14.51 ± 0.21 mg GAE/g DW. Skowyra et al. (2014) reported the content of phenolics in *perilla* leaves was 22.67 ± 0.52 mg GAE/g DW using 50% ethanol for extraction. Hong and Kim (2010) found a value of 12.15 mg GAE/g DW by refluxing extraction with 70% ethanol for 24 hr. The solvent used and the extraction method may affect the yield of polyphenols.

Aqueous and organic solvent extracts of *Perilla frutescens* have been reported to contain considerable amount of phenolic compounds such as gallic acid, isovanillic acid, caffeic acid, ferulic acid, chlorogenic acid, sinapic acid, and rosmarinic acid (Hong, Park, & Kim, 2011). Five main phenolic compounds including caffeic acid, ferulic acid, rosmarinic acid, quercetin, and apigenin were identified in PLE in the present study by comparing the retention time and ultraviolet absorption spectrum of the eluting peaks with those of the polyphenol standards. As shown in Table 1, the content of caffeic acid, ferulic acid, rosmarinic acid, quercetin, and apigenin in ethanolic extracts of *Perilla frutescens* leaf was 4.80, 5.10, 2.95, 6.46, and 3.93 mg/g dry weight (DW), respectively. Recently, polyphenols such as caffeic acid and rosmarinic acid have been demonstrated antioxidant activity, the ability to improve the stability of lipid‐containing foods (Conde, Moure, Domínguez, Gordon, & Parajó, 2011; Medina et al., 2012), and human beneficial effects (Yang, Hong, Lee, Kim, & Lee, 2013). The utilization of *Perilla frutescens* leaf extracts offers the possibility for the preservation of foods and development of functional foods.

**Table 1:** The content of phenolic compounds in PLE

Polyphenolic compounds	Content (mg/g DW)
Caffeic acid	4.80 ± 0.02
Ferulic acid	5.10 ± 0.09
Rosmarinic acid	2.95 ± 0.05
Quercetin	6.46 ± 0.11
Apigenin	3.93 ± 0.04

Note

Values are mean ± *SD* (*n* = 3).

### 3.2 Antioxidant activity of PLE

Antioxidant activity of PLE was assessed by two methods: DPPH radical scavenging activity and ABTS radical scavenging activity. Using two methods provides more comprehensive information on the antioxidant properties of PLE. As shown in Table 2, PLE (1.25 µg/ml to 25.00 µg/ml) exhibited strong DPPH radical scavenging activity (11.2%–78.86%) with an IC_50_ = 12.15 µg/ml. Consistent with our results, Lin et al. found that the methanolic extracts of *Perilla frutescens* leaf at 1.5–25 µg/ml have a scavenging ability of DPPH radicals in the range of 6.7%–63.1% (Lin, Chou, Kuo, & Huang, 2010). Moreover, PLE (1.25 µg/ml to 25 µg/ml) showed great ABTS radical scavenging activity (13.4%–83.72%) with an IC_50_ = 7.26 µg/ml. In another study, 100% scavenging capacity was observed at 300 μg/ml of methanol extract of green perilla leaves (Lee et al., 2017). The strong antioxidant activity of PLE could be caused by the presence of polyphenolic compounds such as caffeic acid, ferulic acid, rosmarinic acid, quercetin, and apigenin which have potent antioxidant activity.

**Table 2:** The DPPH and ABTS scavenging activity of PLE

Concentration (µg/mL)	DPPH scavenging activity (%)	ABTS scavenging activity (%)
	PLE	PLE
1.25	11.25 ± 1.21	13.40 ± 1.24
2.5	25.13 ± 1.67	27.41 ± 2.17
5	36.87 ± 1.45	37.65 ± 1.96
10	42.58 ± 1.79	71.02 ± 3.34
15	63.77 ± 1.86	91.36 ± 3.55
20	77.95 ± 2.24	97.54 ± 3.58
25	78.86 ± 2.19	97.99 ± 3.58

Note

Values are mean ± *SD* (*n* = 3).

### 3.3 Effect of PLE on lipid oxidation of surimi fish ball

The TBARS is widely used as an indicator to assess the degree of lipid oxidation status in food samples (Tokur, Korkmaz, & Ayas, 2006). Phenolic compounds show strong antioxidative activity via quenching of free radical which is believed to initiate lipid oxidation (Schlesier, Harwat, Böhm, & Bitsch, 2002). Plant extracts with high levels of phenolic compounds have been found to inhibit lipid oxidation in chill stored beef patties (Banon, Díaz, Rodríguez, Garrido, & Price, 2007; Jongberg, Skov, Tørngren, Skibsted, & Lund, 2011). In this project, the effect of PLE on TBARS values of surimi fish ball stored at 4°C was investigated. As shown in Table 3, the TBARS values of control samples were significantly higher (*p* < 0.05) than that of PLE‐added (both 0.01% and 0.03%) samples immediately after cooking, indicating that lipid oxidation was effectively inhibited by PLE. The TBARS values were increased significantly (*p* < 0.05) in all samples throughout the entire storage. TBA value was 0.28 mg of MDA/kg sample for control samples at day 0 and reached 0.98 mg of MDA/kg sample at the end of the storage period (days 12). Addition of PLE significantly reduced the rate of increase of TBARS values, especially at concentration of 0.03%, over the same storage period, indicating that PLE could be used as a natural food additive for prevention of lipid oxidation in surimi fish ball. The decreased TBARS values could be attributed to the phenolic compounds which have been demonstrated strong free radical scavenging activities in PLE. Consistent with our results, Lee et al. (2015) found that the addition of *Perilla frutescens* water extract could effectively inhibit lipid oxidation in beef patties.

**Table 3:** Effect of PLE on TBA values (mg MDA/kg sample) of surimi fish ball during cold storage

Days	Control	0.01% PLB	0.03% PLB
0	0.28 ± 0.03^aA^	0.18 ± 0.02^bA^	0.15 ± 0.03^bA^
3	0.46 ± 0.04^aB^	0.33 ± 0.03^bB^	0.26 ± 0.02^cB^
6	0.58 ± 0.05^aB^	0.46 ± 0.04^bB^	0.35 ± 0.03^cB^
9	0.79 ± 0.07^aC^	0.57 ± 0.05^bC^	0.47 ± 0.04^cC^
12	0.98 ± 0.06^aD^	0.72 ± 0.05^bD^	0.65 ± 0.05^cC^

Note

Values are mean ± *SD* (*n* = 3).

Values within treatments in a row with different superscript lowercase letters (a‐c) differ significantly (*p* < 0.05).

Values within storage periods with different superscript numerical (A‐D) in a column differ significantly (*p* < 0.05).

### 3.4 Effect of PLE on protein carbonyl level of surimi fish ball

The formation of carbonyls on protein structure is the main chemical consequence of protein oxidation. The carbonyl content of muscle foods can differ between solubility of proteins, muscle type, and the extent of oxidation (Fagan, Sleczka, & Sohar, 1999). To evaluate the effects of PLE on protein oxidation, the carbonyl content of surimi fish ball without and with PLE was determined during the whole storage. As shown in Table 4, the protein carbonyl contents of all samples increased over the time periods investigated. Compared with PLE‐added samples, samples without PLE gave increasingly higher protein carbonyl contents over the 12 days of storage. In general, the carbonyl content is around 1 nmol/mg protein in nonoxidized muscle tissue, whereas the estimated carbonyl content arranged in 2–14 nmol/mg protein for oxidized muscle tissue, depending on muscle type and the extent of oxidation (Choe, Kim, & Kim, 2017). In the present study, the carbonyl content of PLE‐added samples (both 0.01% and 0.03%) was still < 2 nmol/mg protein until days 9, but the samples without PLE exceeded 2 nmol/mg proteins on days 6, indicating that PLE addition inhibited protein oxidation in surimi fish ball. Consistent with our results, Vuorela et al. (2005) found that plant phenolics addition lowered carbonyl formation in cooked pork meat patties. The addition of antioxidants such as pomegranate rind extract, grape seed extract, and green tea extract at a final concentration of 100 ppb equivalent phenolics significantly reduced protein oxidation of minced fish during frozen storage (Özen & Soyer, 2018).

**Table 4:** Effect of PLE on the protein carbonyl levels (nmol/mg protein) of surimi fish ball during cold storage

Days	Control	0.01% PLB	0.03% PLB
0	1.56 ± 0.07^aA^	1.47 ± 0.06^aA^	1.07 ± 0.08^bA^
3	1.74 ± 0.08^aA^	1.50 ± 0.05^bA^	1.22 ± 0.07^cA^
6	2.22 ± 0.07^aB^	1.93 ± 0.11^bB^	1.67 ± 0.16^bB^
9	3.11 ± 0.11^aC^	2.39 ± 0.16^bB^	2.13 ± 0.16^cC^
12	4.24 ± 0.18^aD^	3.27 ± 0.16^bC^	2.86 ± 0.19^cD^

Note

Values are mean ± *SD* (*n* = 3).

Values within treatments in a row with different superscript lowercase letters (a‐c) differ significantly (*p* < 0.05).

Values within storage periods with different superscript numerical (A‐D) in a column differ significantly (*p* < 0.05).

### 3.5 Effect of PLE on TVB‐N content of surimi fish ball

TVB‐N, mainly composed of ammonia and primary, secondary, and tertiary amines, is produced by the protein degradation due to bacterial and enzymatic actions in the process of fish spoilage. It is an indicator used for quality and freshness evaluation of aquatic products and meat (Yi et al., 2011). The TVB‐*N* values of all samples increased over the duration of storage. The TVB‐*N* values of the control samples increased from 8.71 to 17.83 mg N/kg during the 12 days of storage (Table 5). Compared to the control, the PLE‐added samples (0.03%) had significantly lower (*p* < 0.05) TVB‐*N* values at any time of testing throughout storage, which may be attributed to the antibacterial and antioxidative activities of PLE. Similar finding was documented by Yi et al., (2011) who found tea polyphenols can effectively prevent protein degradation and reduce the TVB‐N content of *Collichthys* fish ball.

**Table 5:** Effect of PLE on the TVB‐*N* values (mg N/kg) of surimi fish ball during cold storage

Days	Control	0.01% PLB	0.03% PLB
0	8.71 ± 0.24^aA^	8.54 ± 0.16^aA^	7.23 ± 0.23^bA^
3	9.17 ± 0.33^aA^	8.62 ± 0.17^aA^	7.99 ± 0.21^bA^
6	11.25 ± 0.58^aB^	10.23 ± 0.25^abB^	9.16 ± 0.46^bB^
9	13.81 ± 0.36^aC^	11.82 ± 0.35^bC^	11.14 ± 0.29^bC^
12	17.83 ± 0.51^aD^	14.87 ± 0.67^bD^	12.53 ± 0.43^cD^

Note

Values are mean ± *SD* (*n* = 3).

Values within treatments in a row with different superscript lowercase letters (a‐c) differ significantly (*p* < 0.05).

Values within storage periods with different superscript numerical (A‐D) in a column differ significantly (*p* < 0.05).

### 3.6 Effect of PLE on microbial load of surimi fish ball

The total plate count and the number of *E. coli*, a pathogenic microorganism, in processed aquatic products of animal origin, lower than 5 × 10^4 ^CFU/g and 100 CFU/g, respectively, as given by the Chinese Standard GB 10136–2015, were generally regarded as the acceptability limit (Kong et al., 2018). As shown in Table 6, no significant (*p* > 0.05) difference was observed in the total plate counts of surimi fish ball among PLE‐added (0.01% and 0.03%) and control samples immediately after cooking. On the 6th day of storage, surimi fish balls without PLE had rapidly growing counts and reached 5.47 × 10^4 ^CFU/g, more than the acceptability limit. PLE‐added samples had lower total plate counts (*p* < 0.05) throughout the storage period, and they did not exceed the limit until 9 days storage. *E. coli* of all samples increased over the duration of storage. The increase of *E. coli* in the control samples is much faster than the PLE‐added samples. The *E. coli* of the control was found at levels greater than 100 CFU/g after 6 days storage. The count of *E. coli* did not exceed the level of 100 CFU/g in samples added with 0.01% and 0.03% until 9 days storage. These results clearly demonstrated that the PLE had strong antimicrobial activity in surimi fish ball and extend the shelf life of surimi‐based food. The antimicrobial activity of PLE was stronger compared to that reported in a previous study in which the addition of water extract of *Perilla frutescens* at a concentration of 0.6% inhibited the growth of *E. coli* O157:H7 in beef patties (Lee et al., 2015), indicating that solvents used for extraction could affect the antioxidant and antimicrobial activities of plant extracts. The final extraction of total phenolic compounds and antioxidant capacity obtained using organic solvents were higher than those obtained with water (El‐Chaghaby, Ahmad, & Ramis, 2014). Phenolic compounds have been reported the principal components responsible for the antimicrobial properties of plants, and they cause morphological changes in pathogens thereby increasing the membrane fluidity and permeability, cause loss of vital intracellular components, and inactivate bacterial enzymes (Lee et al., 2015).

**Table 6:** Effect of PLE on microbiological growth (CFU/g) of surimi fish ball during cold storage

	Days	Control	0.01% PLB	0.03% PLB
Total plate count(CFU/g)	0	75 ± 11^aA^	64 ± 12^aA^	60 ± 15^aA^
	3	(2.61 ± 0.23)×10^4aB^	(1.96 ± 0.32)×10^4bB^	(1.33 ± 0.11)×10^4cB^
	6	(5.47 ± 0.74)×10^4aC^	(3.59 ± 0.58)×10^4bC^	(2.16 ± 0.33)×10^4cC^
	9	(7.83 ± 0.70)×10^4aD^	(6.15 ± 0.24)×10^4bD^	(5.26 ± 0.31)×10^4cD^
	12	(1.49 ± 0.15)×10^5aE^	(1.16 ± 0.13)×10^5bE^	(0.92 ± 0.04)×10^5cE^
E. coli (CFU/g)	0	0^aA^	0^aA^	0^aA^
	3	22 ± 5^aB^	18 ± 6^aB^	7 ± 3^bB^
	6	104 ± 17^aC^	79 ± 15^bC^	35 ± 17^cC^
	9	277 ± 13^aD^	147 ± 18^bD^	89 ± 15^cD^
	12	697 ± 25^aE^	365 ± 16^bE^	214 ± 18^cE^

Note

Values are mean ± *SD* (*n* = 3).

Values within treatments in a row with different superscript lowercase letters (a‐c) differ significantly (*p* < 0.05).

Values within storage periods with different superscript numerical (A‐E) in a column differ significantly (*p* < 0.05).

### 3.7 Effect of PLE on sensory attributes of surimi fish ball

Sensory evaluations of surimi fish ball based on appearance, flavor, texture, taste, and overall acceptability are shown in Table 7. There were no significant differences (*p* > 0.05) among the PLE‐added (0.01% and 0.03%) and control samples immediately after cooking. The sensory attributes of all samples decreased with storage time. However, the scores for each attribute of PLE‐added samples were significantly higher than control samples (*p* < 0.05) at any time during the 12 days of storage. The overall acceptability values were still good in PLE‐added (0.03%) samples after 6 days of storage and fair even after 9 days of storage, while the overall acceptability of control samples was fair after 6 days of storage and poor after 9 days of storage. In another study, beef patties with *Perilla frutescens* water extract also showed higher sensory scores than control samples throughout the storage period (Lee et al., 2015). The decrease in overall acceptability during storage was due to the loss of flavor and texture caused by lipid oxidation, protein degradation, and microbial infestation (Lorenzo, Bedia, & Bañón, 2013). Various volatile flavor compounds in *Perilla frutescens* may have masking effects on undesirable flavors generated in the surimi fish ball (Jung & Lee, 2000). The significant retention of overall acceptability in PLE‐added (0.03%) samples was mainly due to the strong antioxidant and antimicrobial activity of PLE during storage.

**Table 7:** Effect of PLE on sensory attributes of surimi fish ball during cold storage

	Days	Control	0.01% PLB	0.03% PLB
Appearance	0	4.58 ± 0.15^aA^	4.57 ± 0.14^aA^	4.53 ± 0.15^aA^
	3	4.11 ± 0.12^aB^	4.23 ± 0.15^aB^	4.45 ± 0.12^bA^
	6	3.89 ± 0.11^aB^	4.11 ± 0.13^aB^	4.28 ± 0.13^bA^
	9	3.57 ± 0.14^aC^	3.81 ± 0.14^aC^	3.95 ± 0.17^bB^
Flavor	0	4.64 ± 0.10^aA^	4.61 ± 0.16^aA^	4.65 ± 0.12^aA^
	3	4.11 ± 0.14^aB^	4.37 ± 0.13^aA^	4.48 ± 0.16^bA^
	6	3.81 ± 0.11^aB^	3.92 ± 0.14^aB^	4.17 ± 0.12^bB^
	9	2.92 ± 0.15^aC^	3.37 ± 0.13^bC^	3.59 ± 0.18^bC^
Texture	0	4.58 ± 0.13^aA^	4.59 ± 0.16^aA^	4.58 ± 0.15^aA^
	3	4.14 ± 0.12^aB^	4.26 ± 0.11^aA^	4.27 ± 0.16^aA^
	6	3.82 ± 0.17^aB^	4.07 ± 0.15^aB^	4.17 ± 0.14^bB^
	9	3.56 ± 0.14^aC^	3.87 ± 0.12^bB^	3.92 ± 0.14^bB^
Taste	0	4.66 ± 0.12^aA^	4.64 ± 0.14^aA^	4.58 ± 0.12^aA^
	3	4.06 ± 0.15^aB^	4.25 ± 0.13^aB^	4.38 ± 0.14^bA^
	6	3.69 ± 0.15^aC^	3.89 ± 0.14^aC^	4.05 ± 0.13^bB^
	9	2.89 ± 0.13^aD^	3.32 ± 0.17^bD^	3.58 ± 0.16^bC^
Overall acceptability	0	4.64 ± 0.11^aA^	4.61 ± 0.17^aA^	4.65 ± 0.14^aA^
	3	4.05 ± 0.15^aB^	4.16 ± 0.16^aB^	4.31 ± 0.12^bB^
	6	3.61 ± 0.17^aC^	3.92 ± 0.16 ^aB^	4.06 ± 0.14^bB^
	9	2.93 ± 0.14^aD^	3.25 ± 0.15^bC^	3.63 ± 0.13^cC^

Note

Values are mean ± *SD* (*n* = 3).

Values within treatments in a row with different superscript lowercase letters (a‐c) differ significantly (*p* < 0.05).

Values within storage periods with different superscript numerical (A‐D) in a column differ significantly (*p* < 0.05).

## 4 CONCLUSION

Caffeic acid, ferulic acid, rosmarinic acid, quercetin, and apigenin were the main polyphenols in PLE. PLE showed high free radical scavenging activity toward DPPH and ABTS radicals. The addition of PLE in surimi fish balls reduced lipid and protein oxidation and TVB‐*N* values during cold storage. PLE also inhibited microbiology growth of surimi‐based products. In addition, the overall acceptability of PLE‐added (0.03%) samples was higher than control samples during the storage process (*p* < 0.05). Overall, PLE can be considered as a potential ingredient to be used as a natural food additive to improve the shelf life and sensorial qualities of surimi‐based food.

## CONFLICT OF INTEREST

The authors have declared no conflict of interest.

## ETHICAL STATEMENTS

This study does not involve any human or animal testing.
